# Lung Ultrasound in Children with Acute Respiratory Failure: Comparison between Chest X-ray, Chest Computed Tomography, and Lung Ultrasound: A Case Series

**DOI:** 10.5005/jp-journals-10071-23124

**Published:** 2019-02

**Authors:** Shinichi Fukuhara, Yoshimichi Yamaguchi, Yoshiyuki Uetani, Yoshinobu Akasaka

**Affiliations:** 1-4 Department of Emergency and Critical Care Medicine, Kobe Children's Hospital, Kobe City, Japan.

**Keywords:** Lung ultrasound, Acute respiratory failure, Children, Chest X-ray, Chest CT

## Abstract

Respiratory failure is one of the most common and critical problems in pediatric wards. Assessments by chest X-rays (CXRs) are common and prevalent for determining the cause of respiratory failure in children. However, CXRs can be misinterpretated. Some patients may require further evaluation with other tools, such as chest computed tomography (CCT). Lung ultrasound (LUS) has proven useful for detecting lung abnormalities with respiratory failure in adults, but its usefulness in children is still not clear.

We present a series of eight children who were admitted in a tertiary children's hospital. Each child underwent CXR, CCT, and LUS.

In seven of eight cases, both LUS and CCT were able to detect abnormal findings. However, the radiological findings in CXRs were not sufficient. These cases included children with pleural effusion that was comorbid with consolidation, or cases with substantial consolidation that required thoracentesis or proper physical therapy.

**Key messages:**

LUS can be beneficial for evaluating children with respiratory failure that are admitted in pediatric intensive care unit (PICU) and may contribute towards appropriate therapy for children.

**How to cite this article:**

Fukuhara S, Yamaguchi Y *et al.* Lung Ultrasound in Children with Acute Respiratory Failure: Comparison between Chest X-ray, Chest Computed Tomography, and Lung Ultrasound: A Case Series. Indian J of Crit Care Med 2019;23(2):95-98.

## INTRODUCTION

**R**espiratory failure is one of the most common and critical problems for children. Assessments by chest X-rays (CXRs) are common and prevalent for determining the reasons for respiratory failure in children. However, CXR has major limitation. Self-reported CXRs have demonstrated poor sensitivities compared with CCT even though interpreted by radiologist ^1^.

Some patients may require further evaluation with other tools, such as chest computed tomography (CCT). CCT is essential to locate the abnormal regions of lung and is the gold standard to evaluate lung diseases. However, the number of children who need CCT is small for comparison of these three modalities, and the risk of transporting vulnerable patients to another facility to perform the test, and the possibilities of malignancies are problematic.

During the past two decades, lung ultrasound (LUS) has proven useful for detecting lung abnormalities in adults^2^ and recent studies have reported the usefulness of LUS in children with pneumonia^3^ and with bronchiolitis^4^ without evaluation by CCT. In pneumonia, a meta-analysis has shown that the diagnostic accuracy of LUS may be enough to be alternative for CXP^5^. In pediatric pneumonia, there is a report comparing CXR^6^, LUS and CCT, but there is no study in children with acute respiratory failure. In this case series, we compared between portable CXR, CCT, and LUS in children with acute respiratory failure.

## CASE REPORTS

Here we report the radiological results of eight children (Table 1). Ultrasound criteria is as follows. Interstitial syndrome is the presence of multiple B lines including white echographic lung fields with coalescent B lines. Consolidation is a subpleural echo poor or tissue-like structure with blurred margins or wedge-shaped borders which is caused by the loss of lung aeration. Sonographic air bronchograms are hyperechoic or hypoechoic linear or punctiform elements representing air or water in bronchioles that appear within the hypoechoic consolidated lung. Pleural effusion is a hypoechoic or echoic structure with no gas inside. LUS, CXP and CCT were conducted within 24 hours. LUS was conducted by S. F. with more than 5 years experience. CXP was interpreted by pediatric emergency physician, because, in clinical practice in Japan, it is common for chest X-ray images to be interpreted not by radiologists but by physicians involved in the treatment. CCT was interpreted by pediatric radiologist. In seven of eight cases, both LUS and CCT were able to detect abnormal findings. However, the radiological findings in CXRs were not sufficient.

**Figs 1A to C:**
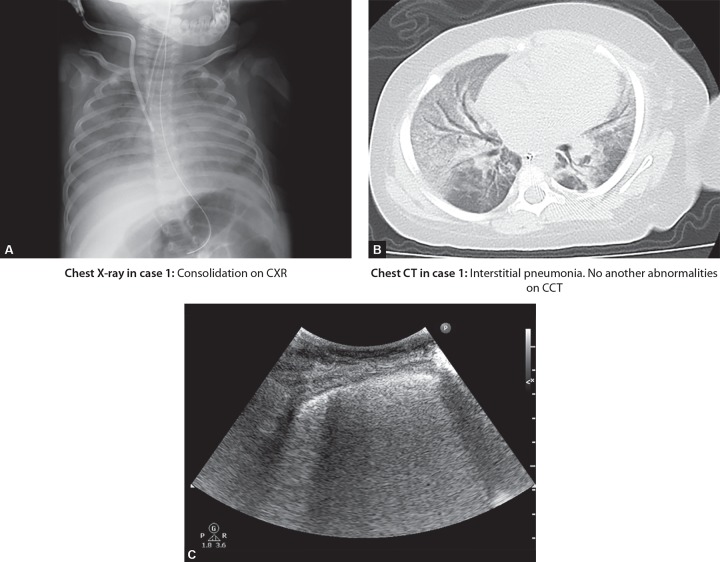
Comparison between chest X-ray, chest CT and lung ultrasound in case 1.

**Figs 2A to C:**
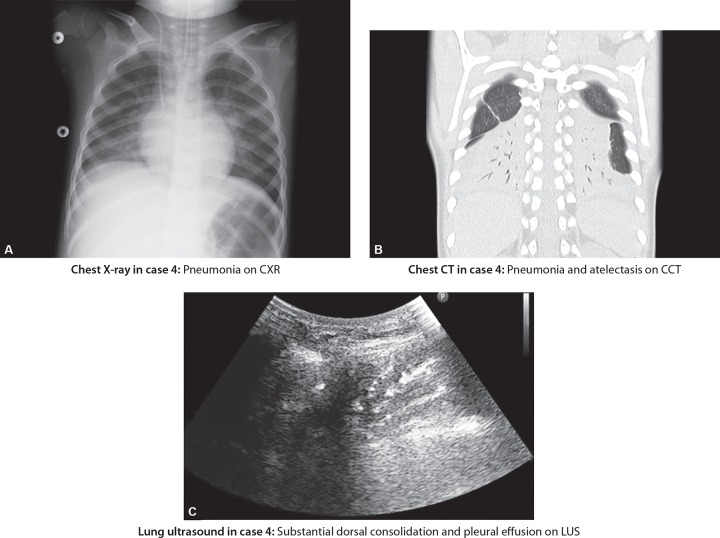
Comparison between chest X-ray, chest CT and lung ultrasound in case 4

In cases 1 and 3 that had interstitial pneumonia or pulmonary edema, the CCT and LUS showed corresponding image findings (Fig. 1, case 1). However, in case 2 with mild interstitial pneumonia with mild symptoms, we were not able to detect the B-lines on LUS. Furthermore, in each of these cases, we could not evaluate the lung region precisely on CXR images. In cases 4 to 6, substantial dorsal consolidations and pleural effusions were found on CCT and LUS images. However, the quantitative evaluation of consolidation, as substantial consolidation, or pleural effusions were not apparent on CXR (Fig. 2, case 4). In case 7, CCT and LUS showed corresponding image findings of mixed abnormalities, consolidation, and pleural effusion. However, the CXR image showed only pneumonia. In case 8, empyema and pneumothorax were detected on LUS, CXR, and CCT.

**Table 1:** Comparison between chest X-ray, chest CT and lung ultrasound

*Case*	*1*	*2*	*3*	*4*	*5*	*6*	*7*	*8*
Age	1 y	2 mo	12 y	4 y	13 y	1 y	7 y	2y
Gender	F	F	M	F	M	M	F	M
Chest X-rays	Pneumonia/	No apparent	Pneumonia	Pneumonia	Pneumonia/	Pneumonia	Pneumonia	Pneumonia
	atelectasis	abnormality			Pleural effusion			
Mechanical	+	- (O_2_ by	- (O_2_ by	+	+	- (O_2_ by mask)	NPPV	+
ventilation		nasal)	mask)					
Chest CT	Interstitial	Mild	Pneumonia/	Atelectasis	Atelectasis/	Pneumonia	Pneumonia/	Pneumonia
	pneumonia	interstitial	Pulmonary	Pneumonia/	Pleural effusion	Consolidation	Atelectasis/	
		pneumonia	edema	Pleural effusion		Pleural effusion	Pleural effusion	
Lung US	B-lines	No apparent	B-lines/	Substantial	Substantial	Substantial	Consolidation/	Consolidation
		abnormality	consolidation	consolidation/pleural effusion	consolidation/pleural effusion	consolidation/pleural effusion	pleural effusion	

### Case 1: Interstitial Pneumonia

A 1-year-old girl diagnosed with pneumonia was under mechanical ventilation in PICU (Fig. 1).

She suffered from acute respiratory failure and her ratio of partial pressure of arterial oxygen to the fraction of inspired oxygen (P/F ratio) was 135 under mechanical ventilation (moderate acute respiratory distress syndrome). Her CXR images revealed pneumonia and atelectasis. However, LUS showed coalescent B-lines and no another abnormalities. CCT revealed only interstitial pneumonia.

### Case 4: Substantial Dorsal Consolidation and Mixed Abnormality

A 4-year-old girl who was on antibiotic treatment and mechanical ventilation was deteriorating on her oxygenation (Fig. 2). Her CXR showed pulmonary opacities and she was diagnosed with pneumonia. One day her oxygenation deteriorated, with unstable oxygen saturation level, and P/F ratio reduced to 135. CXR did not show the source of origin of her worsening hypoxia, thus, the attending doctors decided to conduct CCT. LUS and CCT showed substantial dorsal consolidation, pleural effusion and no other abnormalities. She was prescribed physical therapy including the prone position. After proper therapy, her oxygenation improved, and she was extubated the following day.

### DISCUSSION

Many children suffer from respiratory diseases. However, in critically ill children with respiratory failure, several diverse factors and etiologies of lung diseases configurate comorbid pathologies and rare etiologies may exist. Currently, no clear guidelines exist to evaluate these children. In some cases, misinterpretation and lack of recognition of abnormal regions are inevitable with pediatric CXRs. CXRs demonstrate poor sensitivities compared with CCT even though interpreted by radiologist^1^. CCT is the gold standard method to evaluate lung pathologies, but we have to consider risks of transporting of vulnerable children and risks associated with radiation^2^ which reportedly increases morbidity because of malignancy. The data obtained from study involving adults have shown the usefulness of LUS. Xirouchaki showed that LUS has a significant role on decision making and therapeutic management^7^. Further, Silva reported that cardiothoracic ultrasound may be an attractive complementary diagnostic tool that can contribute in reaching an early therapeutic decision^8^. Ultrasound devices are also safe and available in most pediatric wards; however, the evidence of use of LUS in children with acute respiratory failure is scarce^9–10^. In children with acute respiratory failure, there is no comparison among CXR, LUS and CCT that is regarded as the gold standard. In our single-centre experience, all eight children except one with mild observations on CCT were successfully evaluated with LUS. Compared with CXR, LUS is harmless and sensitive on these children. In our experience, LUS yielded more precise assessments than CXR that can lead to a more accurate diagnosis and management. Further, LUS may be useful for evaluating respiratory failure in children as well as in adults. As with therapy for respiratory diseases, identifying the cause for respiratory failure in children is critical, and thus, the methods must be appropriately altered for accurate identification. LUS, which is considered to have high concordance with CT findings, has the potential to be an alternative modality for CXP in patients with acute respiratory failure. We suggest that LUS should be considered for evaluation in children with acute respiratory failure. However, no studies currently exist that compare CXR, LUS, and CCT. To identify the utility and evidence for the use of LUS in children, further study of LUS is warranted in larger pediatric cohorts.

### CONCLUSION

In our study, including eight cases, LUS showed higher coincidence rate than CXRs compared to the gold standard of chest CT for diagnosis of lung pathology. Further, identical abnormalities in seven of eight children were detected with CCT. We believe that LUS can be beneficial for evaluating children with respiratory failure that are admitted in PICU and may contribute towards appropriate therapy for children.
